# P-1876. Early Exposure to Infectious Diseases Specialty Among Undergraduate Students with Interests in Healthcare Careers

**DOI:** 10.1093/ofid/ofaf695.2045

**Published:** 2026-01-11

**Authors:** Samantha Moreno, Sonja F Tutsch-Bryant, Harlan R Sayles, Jasmine R Marcelin

**Affiliations:** University of Nebraska Medical Center, Yorkville, Illinois; University Nebraska Medical Center, Omaha, Nebraska; University of Nebraska Medical Center, Yorkville, Illinois; University of Nebraska Medical Center, Yorkville, Illinois

## Abstract

**Background:**

Studies investigating early exposure to the Infectious Diseases (ID) workforce have not focused on undergraduate students. We evaluated the experiences, exposure to, and interest in ID of student participants in a college summer enrichment program at a Midwest academic medical center.

**Table 1:**
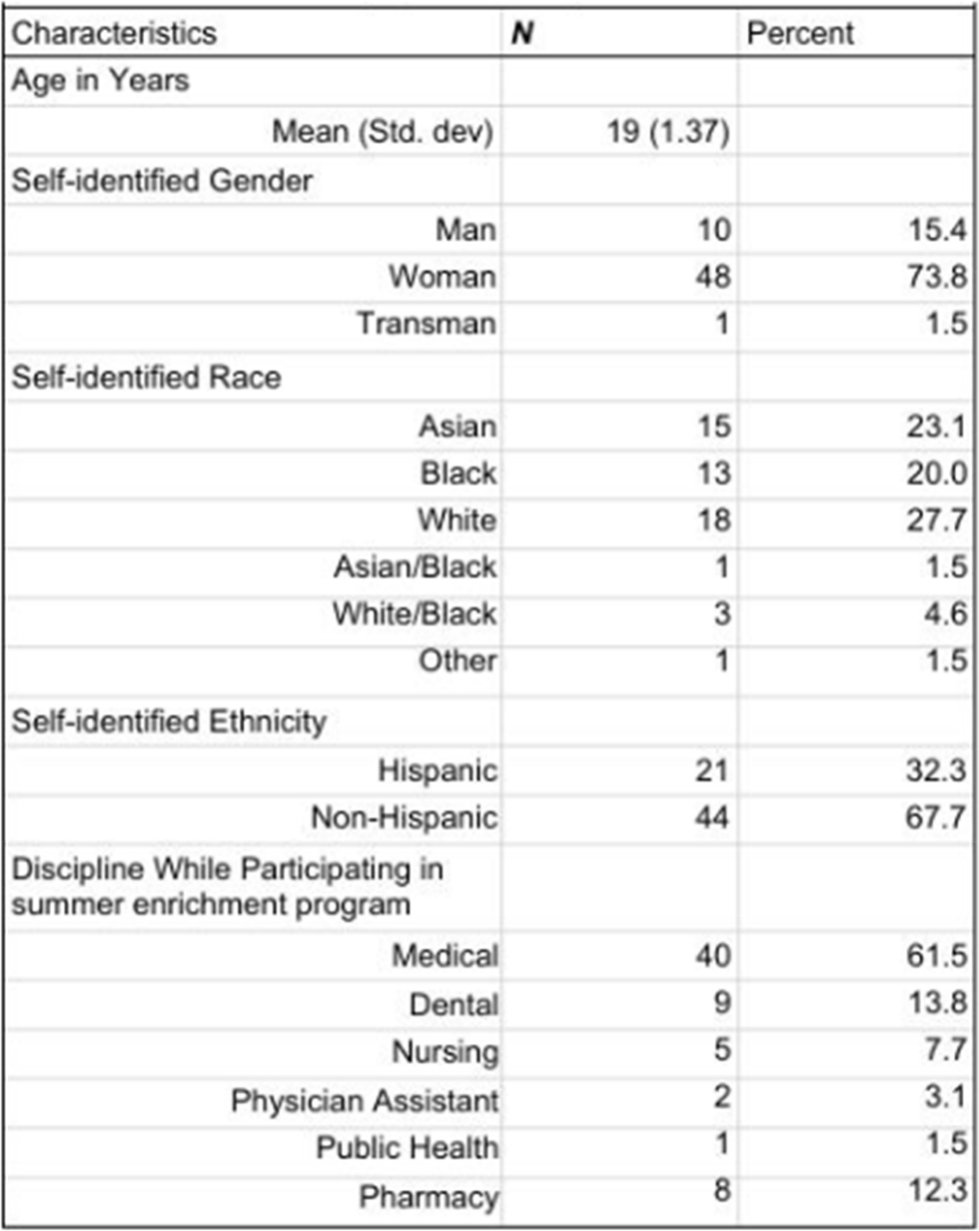
Demographics of respondents

**Figure 1:**
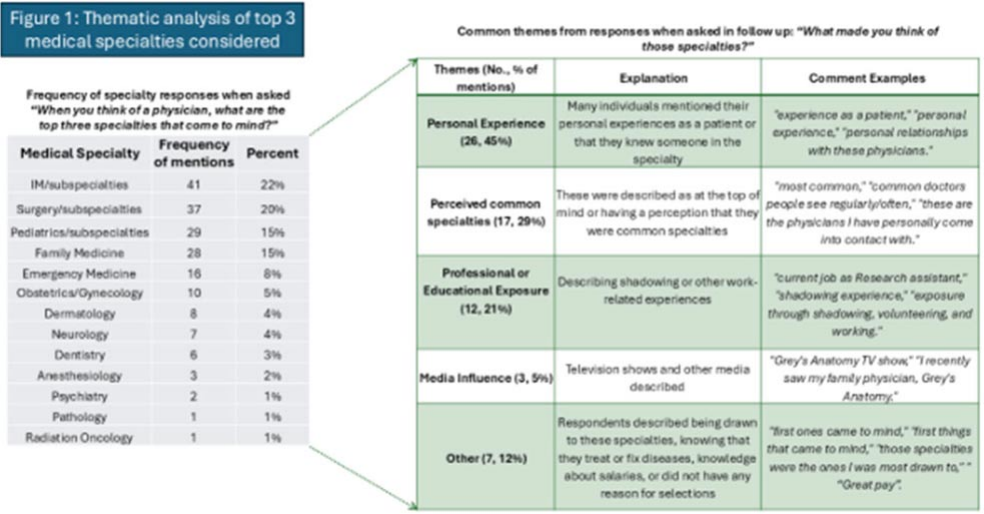
Thematic analysis of top 3 medical specialties considered by students

**Methods:**

A web-based survey was distributed to program alumni who participated from 2019–2024. Survey responses and participant characteristics were summarized using descriptive statistics. Ordinal regression models assessed associations between sources of ID exposure and interest in learning about or pursuing ID as a specialty. Thematic analysis performed on comments.

**Figure 2:**
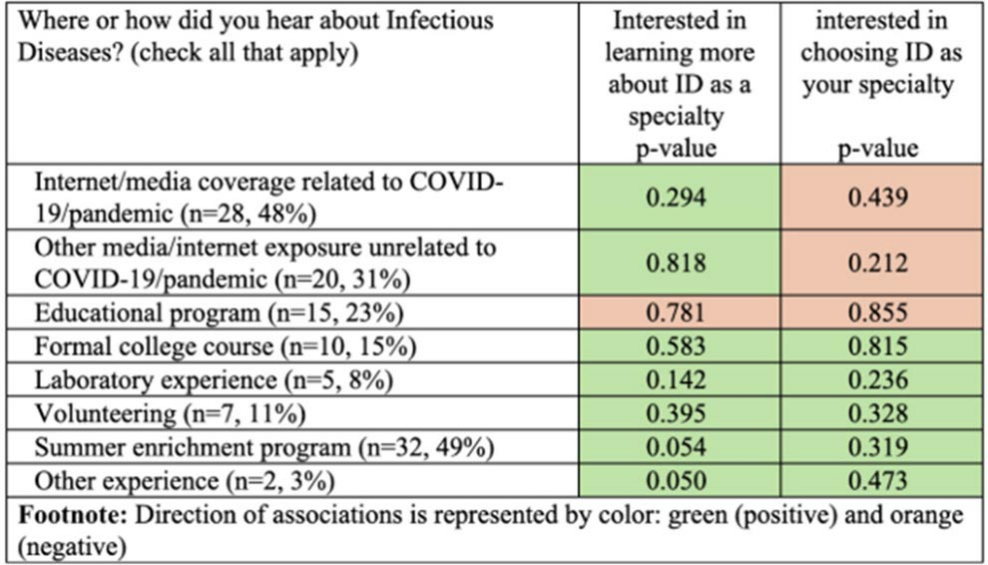
Source of exposure to ID by interest in the specialty

**Figure 3:**
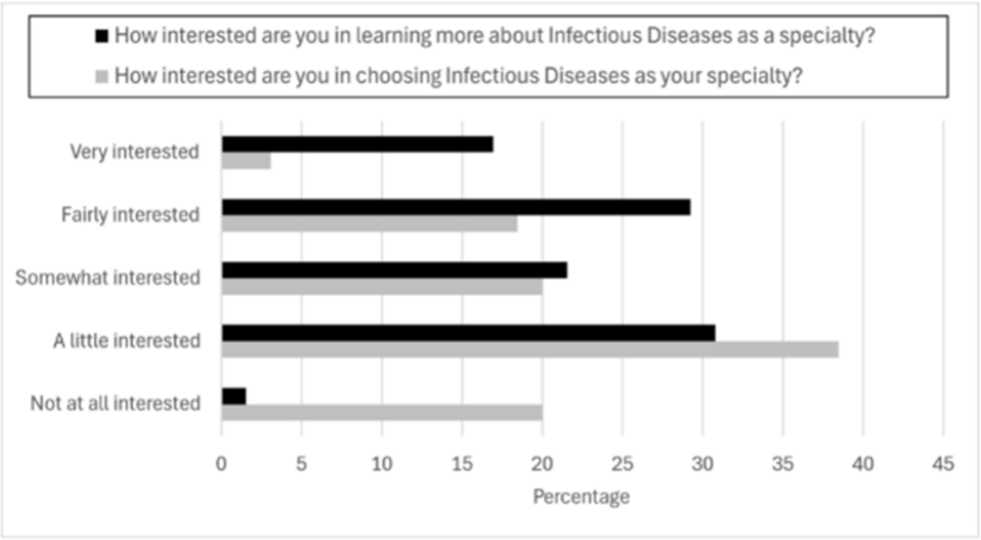
Interest in learning about or choosing infectious diseases as a specialty

**Results:**

Of 446 surveys delivered to program alumni (2019–2024) 65 were completed (14.6% response rate), with higher response rates from the 2022–2024 cohorts (Table 1). Post-program, 63/65 respondents remained interested in healthcare, most commonly medicine (37/65). Thirteen of 32 identifying a specialty of interest cited internal medicine (IM) or pediatrics; 2 named ID specifically. Most students think of IM/subspecialties (22%), surgery/subspecialties (20%), and pediatrics/subspecialties (15%) when medicine is mentioned (Figure 1). The top themes explaining these selections were personal experience (45%), perceived “common” specialties (29%) and shadowing (21%). Eighty percent (52/65) of students were familiar with ID. Among these students, top sources of prior ID exposure included the program (62%), COVID-19-related media (54%). (Figure 2). Direct experience was limited: only 1 student knew a patient and 2 knew a professional in the field. Nearly half (30/65) were at least fairly interested in learning more about ID (Figure 3).

**Conclusion:**

Most students cited the summer enrichment program and consuming COVID-19-related media as their source of exposure to ID. Few students had direct experience with ID, but almost half of students interested in medicine identified interest in internal medicine or pediatrics which could lead to an ID career. This highlights opportunities to increase earlier exposure to the specialty, with shadowing and personal experiences as desirable avenues to develop this interest.

**Disclosures:**

All Authors: No reported disclosures

